# Variation detection based on next-generation sequencing of type Chinese 1 strains of *Toxoplasma gondii* with different virulence from China

**DOI:** 10.1186/s12864-015-2106-z

**Published:** 2015-10-30

**Authors:** Weisheng Cheng, Fang Liu, Man Li, Xiaodong Hu, He Chen, Faustina Pappoe, Qingli Luo, Huiqin Wen, Tian Xing, Yuanhong Xu, Jilong Shen

**Affiliations:** Department of Clinical Laboratory, the First Affiliated Hospital of Anhui Medical University, Hefei, 230022 People’s Republic of China; Department of Microbiology and Parasitology, Anhui Provincial Laboratory of Pathogen Biology and Anhui Key Laboratory of Zoonoses, Anhui Medical University, Hefei, 230022 People’s Republic of China; Department of Blood Transfusion, the First Affiliated Hospital of Anhui Medical University, Hefei, 230022 People’s Republic of China

**Keywords:** *Toxoplasma gondii*, Next-generation sequencing, Variation test, Virulence effectors

## Abstract

**Background:**

*Toxoplasma gondii* is an intracellular protozoan that affects most species of endothermic animals including humans with a great infection rate. The vertical transmission of *T. gondii* causes abortion, constituting a serious threat to humans and leading to great losses in livestock production. Distinct from population structure of *T. gondii* in North America and Europe, Chinese 1 (ToxoDB #9) is a dominant genotype prevalent in China. Among the isolates of Chinese 1, the Wh3 and Wh6 have different virulence and pathogenicity in mice. However, little has been known about their difference at the genomic level. Thus the next-generation sequencing (NGS) approach was used to discover the association of the phenotypical variations with the genome sequencing data and the expression and polymorphisms of the key effectors.

**Results:**

We successfully sequenced the genome of Chinese 1 strains of Wh3 and Wh6. The average sequencing depths were 63.91 and 63.61 for Wh3 and Wh6, respectively. The variations of both isolates were identified in comparison with reference genome of type I GT1 strain. There were 505,645 and 505,856 SNPs, 30,658 and 30,004 indels, 4661 and 2320 SVs, and 1942 and 3080 CNVs for Wh3 and Wh6, respectively. In target search variations of particular factors of *T. gondii*, the dense granule protein 3 (GRA3) and rhoptry neck protein 3 (RON3) were found to have 35 SNPs, 2 indels and 89 SNPs, 6 indels, respectively. GRA3 and RON3 were both found to have higher expression levels in less virulent Wh6 than in virulent Wh3. Both strains of type Chinese 1 share polymorphic GRA15_II_ and ROP_I/III_ with type I, II, and III strains.

**Conclusions:**

Sequencing of the two strains revealed that genome structure of Chinese 1 and type I strains has considerable genomic variations. Sequencing and qRT-PCR analyses of 26 effectors displayed a remarkable variation that may be associated with phenotype and pathogenic differences.

**Electronic supplementary material:**

The online version of this article (doi:10.1186/s12864-015-2106-z) contains supplementary material, which is available to authorized users.

## Background

*Toxoplasma gondii* is an obligate intracellular parasitic Apicomplexa protozoan, parasite which can infect a wide range of hosts and occasionally cause serious diseases in humans [1]. Serological investigations estimated that up to a third of the global population has been exposed to and may be chronically infected with *T. gondii*, although infection rates differ significantly in regions [2]. Infection can result in encephalitis in immune compromised patients, chorioretinitis in immune competent individuals, or congenital transmission if a pregnant woman becomes infected. It has been estimated that, in the absence of effective antiretroviral therapy and immune reconstitution, the risk for development of toxoplasmosis is as high as 30% in a patient with AIDS with positive serologic findings for *Toxoplasma* [3].

The distribution of *T. gondii* genotypes varies greatly with geographical locations [4]. In North America and Europe, *T. gondii* has three clonal lineages that are known as types I, II, and III, comprising the vast majority of isolates. A study has shown that acute virulence to mice with type I strain was uniformly lethal (LD100 = 1), while types II and III were less virulent, with LD50 ≥ 10^3^ and LD50 ≥ 10^5^, respectively [5].

We have previously isolated 51 *T. gondii* strains from animals and human in China. All of the isolates were genotyped at 10 loci by PCR-RFLP. The results showed that the preponderant type of *T. gondii* circulating in China is quite different from those of the clonal lineages or high divergence in the other parts of the world, and 78% of the isolates from animals and humans belong to type Chinese 1 (ToxoDB#9) [6–10].

Wh3 and Wh6 strains displayed a significant phenotypic variation although both possess the identical genotype of Chinese 1. The 10^3^ tachyzoites of Wh3 finally caused all deaths of inoculated mice although 5–7 days later than RH strain, whereas the equal number of parasites of Wh6 gave rise to over 50% survival after 20 days of infection, with a large quantity of cysts observable in the brain tissues [6, 7, 11]. In Asia, genotypes Chinese 1 dominates, which is in stark contrast to the other continents of the world [12]. No data yet on genome of Chinese 1 *T. gondii* and its genome variation have been reported compared to type I strain although the 9–10 alleles-based structure and mouse virulence have been explored [6–8, 13–15]. To achieve this, we used the next-generation sequencing (NGS) platform for whole genome analysis of virulent Wh3 and less virulent Wh6 strains to study the genomic diversity between type I GT1 and the Chinese 1 Wh3 and Wh6 strains with different virulence.

## Methods

### Mice

Female Swiss Webster (SW) mice (specific pathogen free) aged 6 to 8 weeks were obtained from Anhui Laboratory Animal Center, China. The mice were treated in compliance to the Guiding Principles for the Care and Use of Research Animals established by the Anhui Medical University, China (Approval No. AMU26-081108).

### Parasites

Wh3 and Wh6 strains of *T. gondii* were routinely maintained in the laboratory by *in vivo* passage in mice. Five female Swiss Webster (SW) mice received an intraperitoneal (i.p.) inoculation of 10^4^ tachyzoites obtained from the Wh3 strain pre-infected mouse. Brain tissues were collected from the Wh6 strain infected mice, and homogenized in 5 ml of 0.9% (W/V) saline containing antibiotics (penicillin 1000 U/ml, streptomycin 100 μg/ml). The brain tissue homogenate containing 400 cysts was inoculated into each of the 10 mice i.p. . Dexamethasone at a dosage of 2.5 mg was administered to each mouse in the first 3 days after cysts inoculation. When obvious clinical manifestations were observed, the mice were euthanized and introduced into 75% ethanol for 30 s. The ascitic fluid of the infected mice was collected and the tachyzoites were purified as previously described [16].

### DNA extraction, libraries construction and sequencing

Genomic DNA of the Wh3 and Wh6 strains of *T. gondii* were extracted separately from the pre-purified tachyzoites using QIAamp® DNA Mini kit (QIAGEN, Germany) following the manufacturer’s protocol for cultured cells. Using the Covaris ultrasonic processor (Covaris, USA), DNA samples were randomly sheared to ~500 bp in size. Fragmented DNA was end-repaired using T4 DNA polymerase and an ‘A’ base was added to the ends of double strand break DNA. Next, DNA adaptors (Illumina, USA) with a single ‘T’ base overhang at the 3’ end were ligated to the above products. These products were then separated on an agarose gel, excised from the gel, and purified. The adaptor modified DNA fragments were enriched via PCR amplification using Illumina paired-end PCR primers (Illumina, USA). The concentration of the libraries was initially measured by Qubit®2.0 (Life technologies, USA). The libraries were diluted to 1 ng/μl and the Agilent Bioanalyzer 2100 (Agilent, USA) was used to test the insert size of the libraries. In order to ensure their quality, SYBR green qRT-PCR protocol was used to accurately dose the effective concentration of the libraries. The libraries were sequenced on the Illumina HiSeq 2000 platform (Illumina, USA) by Novogene Bioinformatics Institute, Beijing, China.

### Filtering reads and mapping reads

Paired end (PE) reads with 125 bp were determined and the clean reads were collected from sequenced reads, which were pre-processed to remove adaptors and low quality paired reads. The following criteria were used to remove the low quality reads: i) containing more than 10% ‘N’s; ii) more than 50% bases having low quality value (Phred score < = 5), with alignment of the clean reads of each strain to the *T. gondii* GT1 genomic reference v12.0 using the Burrows-Wheeler Aligner (BWA) [17], and iii) duplicated reads were removed and coverage values were calculated using SAMTOOLS [18].

### Variations identification and annotation

Single nucleotide polymorphisms (SNPs) and insertions/deletions (indels) (<50 bp) were calculated and identified with MPILEUP in SAMTOOLS [18]. In order to reduce the SNPs detection error rate, we filtered the SNPs in which the supported reads number was less than 4 or quality value was less than 20. The bam file produced from the mapping procedure was analyzed for structural variations (SVs) detection by BreakDancer [19] with default parameters. SNPs, indels and SVs were displayed using Savant Genome Browser [20]. Copy number variations (CNVs) were detected with CALL in CNVnator [21]. Functional annotation of all the genetic variants was completed by ANNOVAR [22]. And the Circose plots were created by using Circos [23].

### qRT-PCR detection

Total RNAs of tachyzoites from type I strain RH, Wh3 and Wh6 strains were extracted using TRIzol reagent (Invitrogen, USA) and reverse-transcribed with RevertAid First Strand cDNA Synthesis Kit (Thermo Fisher, USA). Primers for qRT-PCR were designed using Primer 5.0. The primers for qRT-PCR were presented in Additional file 1: Table S1. LightCycler® 480 SYBR Green I Master (Roche, Germany) was used in the qRT-PCR experiments and all reactions were performed on Cobas Z480 system (Roche, Germany). To quantitate gene relative expression, the comparative *C*_T_ method was adapted in this study [24]. Statistical analysis of the data was performed using One-way ANOVA test (Dunnett-*t* test). Significant differences were considered at *P* < 0.05.

## Results

### Sequencing results

The Wh3 and Wh6 were sequenced using Illumina HiSeq 2000, and all the data were subjected to quality assessment to obtain the clean data (Tables 1 and 2). The sequencing yielded 11.287 GB of raw data, 11.195 GB of clean data were obtained after filtration. The sequencing quality was considered to be appropriate and the data were sufficient for variation detection. Two samples sequencing reads were aligned to the *T. gondii* GT1 genomic reference v12.0. The mapping rate of Wh3 sequencing data was 94.84% and that of Wh6 was 59.82%. Average genome coverage depth of both samples were over 63× (Tables 1 and 2).

**Table 1:** Statistics of Wh3 and Wh6 sequencing

Summary of sequencing data quality							
Sample ID	Raw bases (bp)	Clean bases (bp)	Effective rate (%)	Error rate (%)	Q20 (%)	Q30 (%)	GC content (%)
TgCtWh3	4343824750	4320993000	99.47	0.06	91.69	85.56	52.27
TgCtWh6	6942732250	6873966500	99.01	0.04	94.06	89.09	48.39

**Table 2:** Statistics of Wh3 and Wh6 mapping

Summary of sequencing depth and coverage						
Sample ID	Mapped reads	Total reads	Mapping rate (%)	Average depth (X)	Coverage 1X (%)	Coverage 4X (%)
TgCtWh3	32785657	34567944	94.84	63.91	98.77	97.88
TgCtWh6	32898129	54991732	59.82	63.61	98.88	98.01

### SNPs and indels calling

**Fig. 1:**
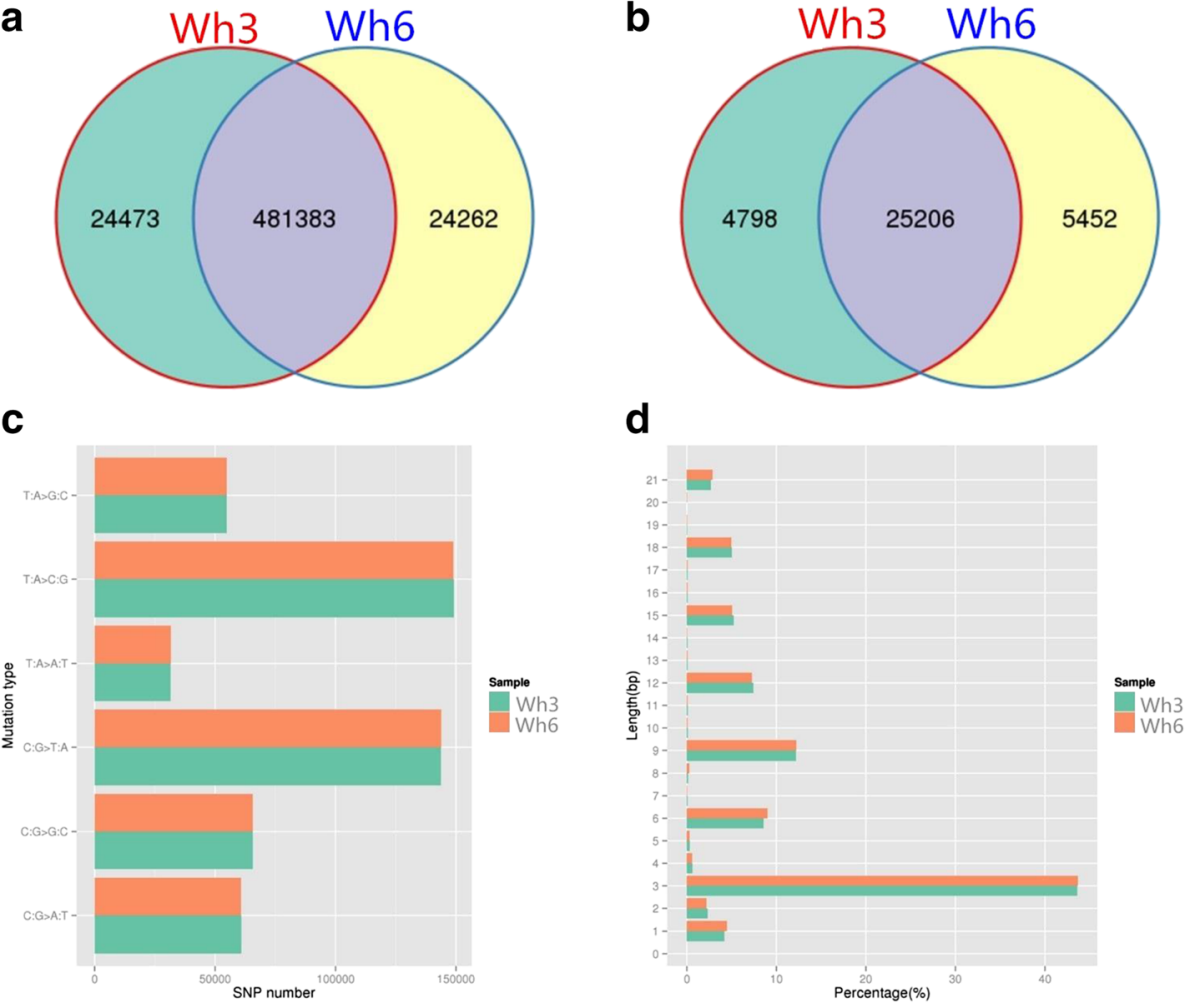
SNPs and indels comparison and distribution. **a** Venn diagram of shared and unique SNPs between two samples. **b** Venn diagram of shared and unique indels between two samples. **c** SNP mutation type distribution of Wh3 and Wh6. **d** CDS indels length distribution of Wh3 and Wh6

SNPs and indels calling for two samples were carried out using the *T. gondii* GT1 genome as a reference (TGGT1 version 12.0; ToxoDB.org) [25]. The SAMTOOLS [18] was used for the detection of SNPs and indels and filter out the poorly supported SNPs. The Wh3 contained 505,856 SNPs and 12,522/17,482 small indels, while the Wh6 contained 505,654 SNPs and 12,793/17,865 small indels. Statistics of shared and unique SNPs and indels of each sample are shown in Fig. 1, indicating that 117,077 SNPs from the Wh3 and 116,952 SNPs from the Wh6 were located in exonic regions, and 61,706 and 61,701 SNPs from Wh3 and Wh6 were non-synonymous. One hundred and forty-five SNPs of the Wh3 and 140 SNPs of the Wh6 were Stop gain which led to early termination of gene expression. Twenty-eight SNPs of them belong to the type Stop loss which may cause delay termination of gene expression or inability of the gene expression to terminate. Summary of all annotation data of SNPs and indels is listed in Additional file 2: Table S2. The distribution of SNP mutation type and CDS indels length was also detected. Type T: A change to C: G and type C: G change to T: A constituted majority of the SNPs, and 3 bp indels accounted for more than 40% of both samples. All the other data of the distribution can be seen in Fig. 1.

By analysis of the unique variations of two samples, we found that the unique variations of SNPs and indels were located in 2847 and 2452 genes for the Wh3 and 2868 and 2613 genes for the Wh6 except for the intergenic annotation position. And by analysis of the exonic variations, the mutations were located in 992 and 268 genes for the Wh3 and 1008 and 243 genes for the Wh6 (Tables 3 and 4).

**Table 3:** Summary of annotation data of unique variations containing SNPs and indels

Summary of annotation data of unique variations of SNPs and indels									
	Downstream	Exonic	Intergenic	Intronic	Splicing	Upstream	Upstream; downstream	5’UTR	Total
Wh3-SNPs	2300	4529	6445	9049	9	2013	127	1	24473
Wh6-SNPs	2312	4405	5936	9500	1	1951	154	3	24262
Wh3-indels	530	345	600	2877	4	418	24	0	4798
Wh6- indels	599	297	616	3462	6	434	38	0	5452

**Table 4:** Summary of comparing data of genes containing SNPs and indels

Comparing data of genes containing SNPs and indels			
	Same	Wh3 unique	Wh6 unique
SNPs (except intergenic)	2094	753	675
Indels (except intergenic)	1938	514	774
SNPs (exonic)	646	346	362
Indels (exonic)	167	101	76

### SVs and CNVs

**Fig. 2:**
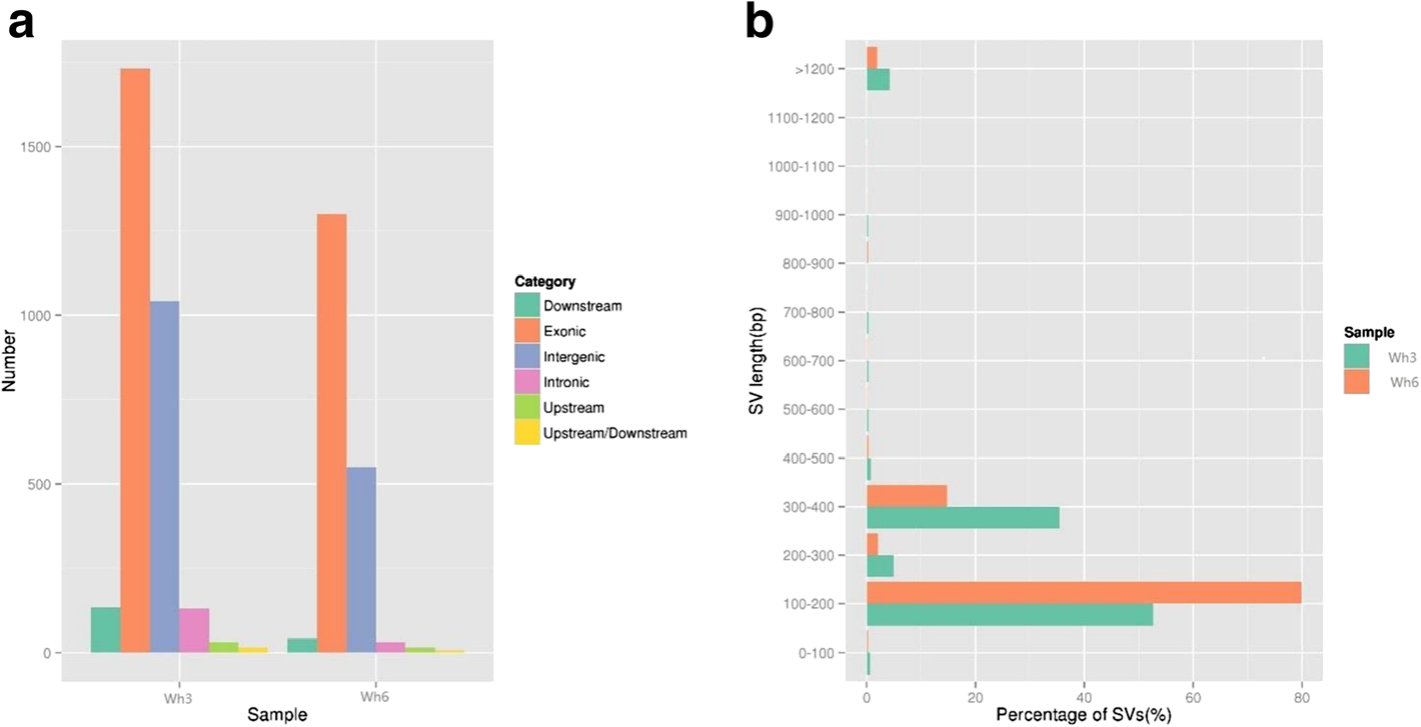
CNVs and SVs distribution. **a** CNVs annotation results distribution of two samples. **b** SVs length distribution of two strains

Structural variations like insertion, deletion, inversion, intra-chromosomal translocation (ITX), and inter-chromosomal translocation (CTX) were detected using BreakDancer [20]. The SVs, which have less than two supported PE reads, were filtered out. The Wh3 contains 2320 SVs compared to GT1 reference genome, with 668, 739, and 35 of insertions, deletions and inversions, respectively. The Wh6 strain, however, contained 4661 SVs, of which 3132 were insertions, 765 were deletions and 25 were inversions. The statistics of SVs annotation results are seen in Tables 3 and 4. The distribution of SVs length revealed nearly 80 % SVs from Wh3 and over 50 % SVs from Wh6. The statistics of SVs annotation results is presented in Additional file 2: Table S2. The lengths range from 100 to 200 bp. The distribution of other length is exhibited in Fig. 2.

CNV including copy number deletion and duplication, potential duplications and deletions were determined through the genome coverage depth of different reads. For the sample Wh3, the duplication number and length were 85 and 282,700 bp, respectively; and the deletion number and length were 2995 and 4,940,000 bp, respectively. However, the Wh6 contains 90 duplications and 1852 deletions, with a deletion number of far less than Wh3, and a much longer deletion length (7,157,700 bp) than Wh3. Its duplication length, however, contains 328,800 bp. The statistics of CNVs annotation results is shown in Additional files 2: Table S2. The distribution of CNVs indicated that majority of CNVs were in the exonic region. All variations in the genome are displayed in Fig. 2.

**Fig. 3:**
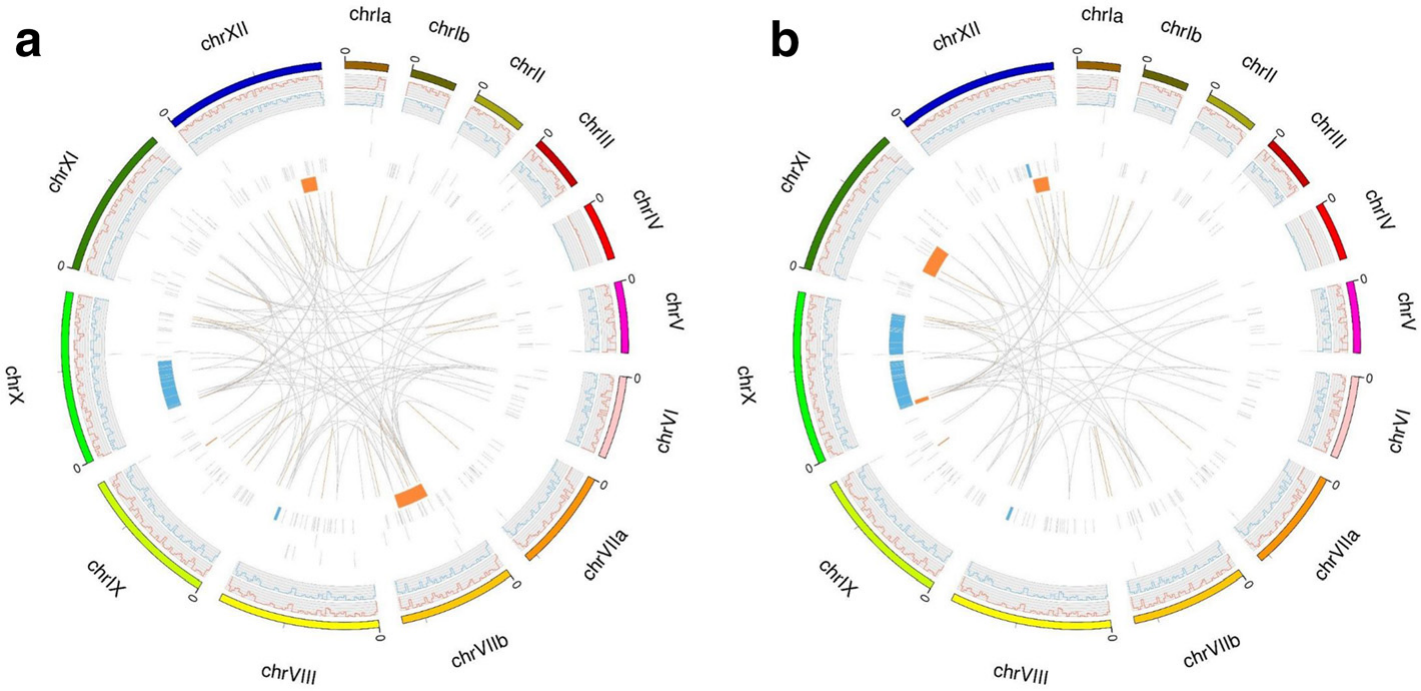
Circos plots of of two samples genome variations. **a** Variation Distribution of Wh3. **b** Variation Distribution of Wh6. For the InDels and SNPs, show density distribution in chromosomes; for the SV and CNV, show their location and size in chromosome. And the figures from the outside to the inside as follows: chromosome, SNP, indel, CNV duplication, CNV deletion, SV insertion, SV deletion, SV inversion, SV ITX, SV CTX

Four kinds of unique variations of Wh3 and Wh6 are listed in Additional file 3: Table S3 and Circose plots of all the genome variations are shown in Fig. 3.

### Key effectors chosen and variation searching

In order to compare the genomic difference between Wh3 and Wh6, coding genes of a group of virulence-/invasion-associated effectors like rhoptry proteins (ROPs), dense granule proteins (GRAs), microneme proteins (MICs), rhoptry neck proteins (RONs), surface antigens (SAGs), etc. were subjected to sequencing and polymorphic comparison. All the genes of interest and the variations are listed in Additional file 4: Table S4. Among them, GRA3 and RON3 were found to have more SNPs and indels compared with the others. The GRA3 contained 35 SNPs and 2 indels, and RON3 contained 89 SNPs and 6 indels in comparison with the reference strain.

**Fig. 4:**
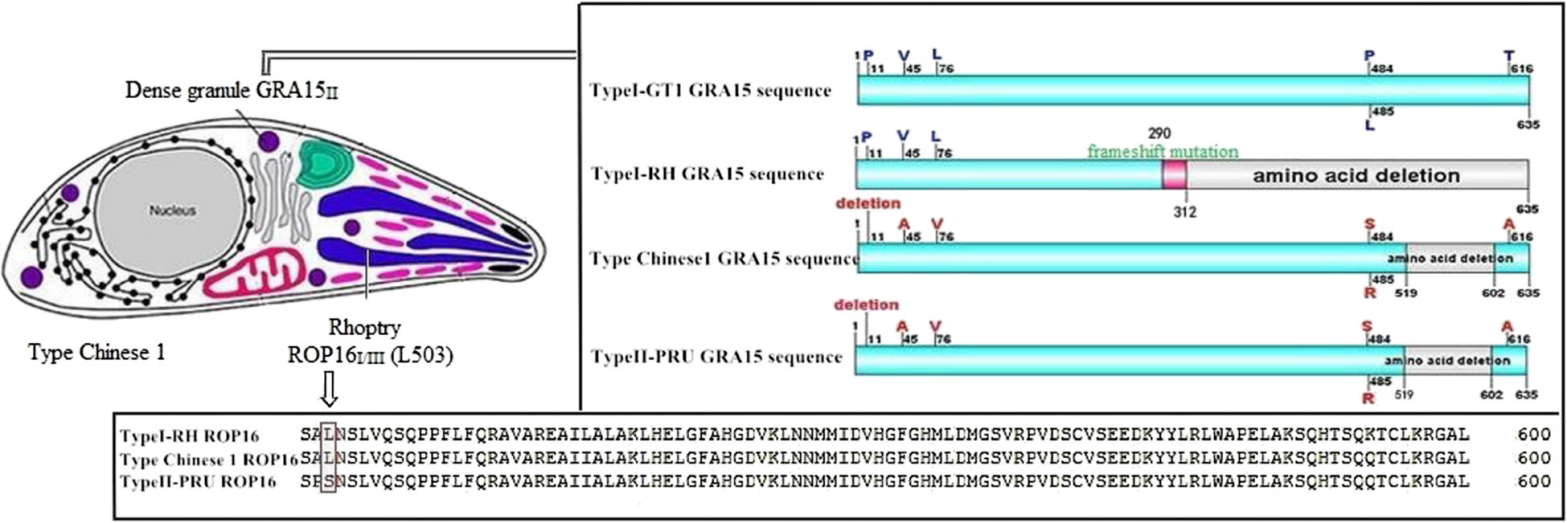
Alignment of ROP16 and GRA15 sequences between type I, type II and type Chinese 1

Interestingly, both strains share the polymorphic 503L of ROP16_I/III_ and GRA15_II_ compared with type I, II, and III although a complete homology of ROP16 and GRA15 was noted between the Wh3 and Wh6 strains (Fig. 4).

### qRT-PCR detection

**Fig. 5:**
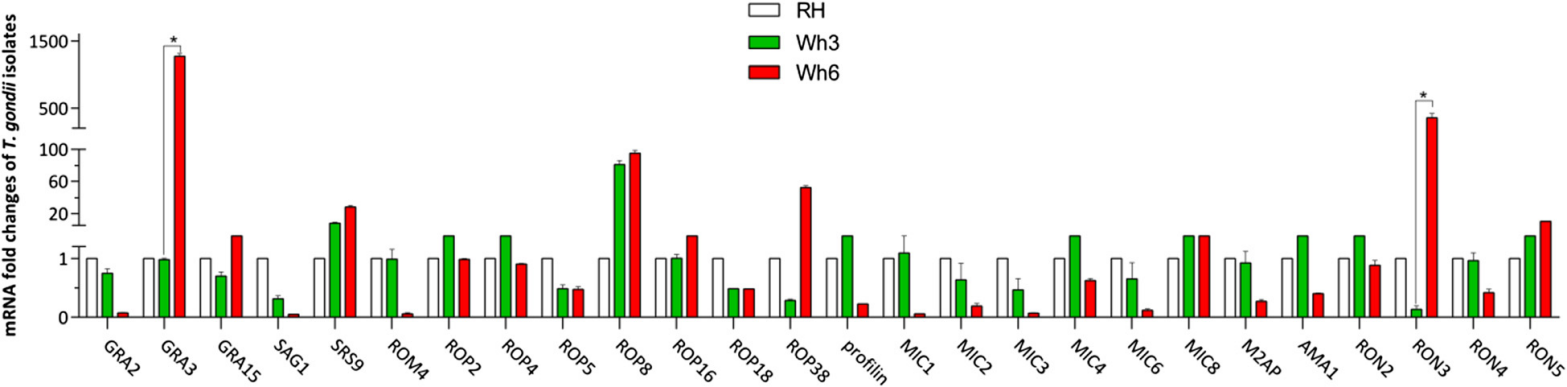
Gene expression profiles among the three strains. It shows a significant increase of RON3 and GRA3 expression in Wh6 strain. Values were reported as means of triplicate test with error bars indicating stardard deviation

In order to find the genes which are associated with the variable phenotypes including virulence in the two strains of type Chinese 1, the gene profile of Wh3, Wh6 and RH was detected by qRT-PCR (Fig. 5). Significant overexpression of GRA3 and RON3 and under expression of ROM4, profilin, M2AP, AMA1, RON2, RON3, RON4 were observed in less virulent Wh6 when compared with virulent Wh3 (*p* < 0.05). High expressions of SRS9, ROP8, MIC8, and RON5 and low expressions of SAG1, ROP5, and ROP18 were seen in the Wh3 and Wh6 strains compared with the RH strain, whereas no significant difference was noted between Wh3 and Wh6.

## Discussion

*Toxoplasma gondii* is arguably the most successful protozoan which is known to have ability to subvert the host cells behavior [26]. Recent studies indicate that *T. gondii* populations in different regions of the world have their own independent evolution until recently. Ships populated by rats, mice, cats, and other animals provided unprecedented opportunities for migration of *T. gondii* [4] and sexual recombination, especially in South America, plays an important role in shaping *T. gondii*’s genetic diversity [27]. Unlike the several archetypal lineages in North America and Europe, and a high diversity in South America, *T. gondii* in Asia appears to have a high degree of genetic uniformity. The genotype Chinese 1 is by far the most commonly found in China mainland, accounting for 50 ~ 78% of the isolates [6, 7, 9, 28] although other genotypes are also noted. In this study, two strains of Chinese 1, Wh3 and Wh6 with different virulence to mice, were subjected to sequencing. The purpose of our study was to explore the genomic differences between Wh3, Wh6, and GT1 by the next-generation sequencing technology. Additionally, the gene differential expression of the key effectors was investigated that are associated with strain virulence and host modulation.

In comparison with type I strains (GT1 or RH-ERP), Wh3 and Wh6 contained more abundant variations. The SNPs and indels of the two isolates distributed in more than 2000 genes, the unique exonic mutations of SNPs and indels, however, existed in 346 and 101 genes for Wh3 and 362 and 76 genes for Wh6, respectively.

Yang et al. compared the genetic difference of type I strain of GT1 with RH-ERP (subcloned RH strain by Elmer Pfefferkorn) [29, 30]. A total of 1394 SNPs and indels were identified and 230 SNPs/indels were within the predicted coding regions [30]. Comparison of the spontaneous mutations of three lab-strains and chemically induced mutations of *T. gondii* revealed that spontaneous SNPs located in non-coding regions or were synonymous mutation to protein coding and tended to have a transition *vs* transversion ratio (ts/tv) of 0.91, lower than chemical induced strains [31]. The ratio of ts/tv in the present two samples, however, were 1.375 and 1.374, respectively, significantly higher than that in chemical induced strains.

Additionally, we carefully analyzed the molecular effectors that have been believed to be closely associated with *Toxoplasma* virulence and its interactions between parasites and host cells. ROP5 together ROP18 was reported to be able to enhance the virulence of *T. gondii* [32, 33]. Correspondingly, the expression of both effectors in Wh3 and Wh6 was found to be remarkably lower than that in type I strain although neither genetic variation in exon regions nor expression of ROP5 and ROP18 was noted between the two strains. In addition, we found that the transcriptional level of ROP38 of less virulent Wh6 was obviously higher than that of virulent Wh3 and RH strains, which coincides with the previous report that ROP38 was up-regulated in low virulence strains [34]. Dense granule protein 3 (GRA3) is known to be secreted by the parasites after invasion and directly inserted into the parasitophorous vacuole membrane (PVM) as a soluble [35] and oligomeric protein and plays a role in the acute infection phase of type II strains, but not essential for *in vitro* culture [36]. The significance of dramatically higher expression of GRA3 in less virulent Wh6 than in virulent Wh3 and RH remains unknown although it has been believed to interact with host cell CAML [37] and induce anti-apoptosis [38].

The MIC3, 4, 6 and ROM4 play synergistic parts with MIC1 in tachyzoite invasion [39–41]. We noted that the expression levels of all these genes were generally lower in less virulent Wh6 than in virulent Wh3 and RH. The profilin and MIC2 and its associated protein M2AP are essential for gliding motility and host cell invasion [42–45], both of them here showed a low expression. RON3 helps for tachyzoites invasion into host cells with assistance of RON2 and RON4 [46]. RON2, 4, 5 and AMA1 are the components of the moving junction complex [47]. In agreement with this, the expression of these effectors in the Wh6 strain was lower than that in the RH and Wh3 strains except for RON5, suggesting a putative cause of relatively low invasion capability of Wh6.

More interestingly, different from type I/III and type II strains, both Wh3 and Wh6 of type Chinese 1 have the distinguishing features of polymorphic ROP16_I/III_ at the amino acid of 503L (leucine), which is identical to the type I GT1 strain. ROP16_I/III_ has been defined to be able to directly phosphorylate STAT3 and STAT6 and drive alternatively activated macrophage differentiation (M2), leading to acute death by excessive parasite burden [48–50]. Additionally, the polymorphic GRA15_II_ was also noted in the two strains of Chinese 1 that contains a deletion of 84 amino acids between 519 and 602 that is consistent with type II ME49 strain. The type II strain-associated GRA15_II_ is believed to strikingly activate NF-κB pathway, resulting in a significant classically activated macrophage (M1) polarization in host innate immunity against *Toxoplasma* infection [51]. It has been reported that, in a type II GRA15_II_ background, a type I copy of ROP16_I/III_ may still significantly inhibit NF-κB activation [51], which may account for the fatal feature of Wh3 strain in mice. We also aligned the sequence of GRA15 and ROP16 of both Wh3 and Wh6 strains, and found that GRA15_II_ were predominant in all strains examined except for XZ7 (ToxoDB#205) strain which showed a GRA15_I_ identical to type I GT1 (data not published). More importantly, we tested the isolates collected from animals (stray cats) for ROP16 and found that all parasites, except for XZ9 and XZ37 isolates, possess the virulent ROP16_I/III_ (data not published), which is quite different from the report by Alvarez that ROP16 nucleotide sequences from patients may be clustered with mouse-virulent strains (83.3%), whereas ROP16 nucleotide sequences from meat samples (animals) may be clustered with mouse-avirulent strains (100%) [52].

## Conclusions

Next-generation sequencing of type Chinese 1 *Toxoplasma* revealed a large number of variations between the virulent Wh3 and less virulent Wh6 strains when compared with the reference strain of type I GT1 strain. The strains of Wh3 and Wh6 share the polymorphic virulent ROP16_I/III_ and avirulent GRA15_II_, and the mouse-virulent ROP16 nucleotide sequences (ROP16_I/III_) were found in all Chinese 1 strains collected from animals (cats), suggesting that, different from the parasites of other continents of the world, strains of type Chinese 1 might have the distinct pathogenicity and immune response process to the host including humans due to their unique features of ROP16_I/III_ and GRA15_II_.

## Additional files

Additional file 1: Table S1. Primer for qRT-PCR. (DOCX 15 kb) Additional file 2: Table S2. A: Summary of annotation for SNPs; B: Summary of annotation for indels; C: Summary of annotation for SVs; D: Summary of annotation for CNVs. (DOCX 18 kb) Additional file 3: Table S3. Unique variations of Wh3 and Wh6. (XLSX 4419 kb) Additional file 4: Table S4. All the chosen genes and variations in those gene. (XLSX 26 kb)
