# Supplementary material for: Variation detection based on next-generation sequencing of type Chinese 1 strains of Toxoplasma gondii with different virulence from China
Source: BMC Genomics. 2015 Oct 30;16:888. doi: 10.1186/s12864-015-2106-z (PMC4628340; doi:10.1186/s12864-015-2106-z)
Supplement: Additional file 1: Table S1. — Primer for qRT-PCR. (DOCX 15 kb) [file 12864_2015_2106_MOESM1_ESM.docx]

Table S1 Primer for qRT-PCR

| Gene | Sense | Anti-sense | Length |
| --- | --- | --- | --- |
| ROP2 | CTCAACACACAAGTCCCTCGT | CATTCGTTCCGCTGTAGTCTC | 244bp |
| ROP4 | GAAGCCATTAGTTCAGAAAGC | ATGAGTTGGAGGAAGCAGA | 214bp |
| ROP5 | CACTATGGGTGCCGAGAAT | CTGAGACTGAACTGCCACAAC | 151bp |
| ROP8 | GCGGGTCTCCACCACTAT | CGTCTCGCACCAGATGTT | 112bp |
| ROP16 | TCCAGGTAGCAGGCATAGAA | CAGCACCGAGACCTACTTCA | 107bp |
| ROP18 | GTTAGAGGGACAGCCAGCAT | TGACAGACGCATCTCCAAAT | 113bp |
| ROP38 | ATCTGTGCGTGTTGGGTT | GCATCGCCTTCTGGAGTTA | 114bp |
| GRA2 | CAGCGGTAAACCTCTTGATG | CGGTTCTTCTGGCTCTTGTT | 94bp |
| GRA3 | AAGCGGCTCATTCCATTTCT | ACACCCGTAACTGGTTCTGC | 119bp |
| GRA15 | GCTTGGGTATCTTACGGTGCT | CGTTTTCGGTCTGTTCACTCT | 242bp |
| GAPDH | TAAGTTCGGCATCGTTGAG | GGAAGGAATAATCTTGCCCACA | 178bp |
| SAG1 | GTCGTTCTTGCGATGTGG | TTTGCCTGTTGGGTGAGTA | 188bp |
| SRS9 | GGCGTGAGTTCCAAGGTA | TCCAGCCAGAGGATTAGG | 189bp |
| MIC1 | TGAAGTTGAAGTCTGCCTACC | GACTGTTCCTTACCGCCAC | 159bp |
| MIC2 | AGAGCGGCAAAGGAGATT | CCAGTCAGCCCGAAGATA | 153bp |
| MIC3 | GCGACGGCGAAACTCTA | GCAGGTGTAGCCATTCTCAT | 134bp |
| MIC4 | CCGTTTGGTTTGGAGTGG | GGAGCCCTTGATCCCTTA | 187bp |
| MIC6 | AGGGAAGATGAAGGTGAAAGT | ACCTCGGCTCTGCTGACT | 135bp |
| MIC8 | TTGCGGACCACCCTCTAT | CACCGACCGTTCCTCACT | 96bp |
| RON2 | CGACAGCCAACCAGCACA | GGTAGGCAATCATCAGGGTCA | 117bp |
| RON3 | AGGACTGGAAGAACCCTCGT | CGCTTTAGAATGGCTTCCAG | 109bp |
| RON4 | AGTCCTGCGTGAAATACATAAAG | TTCCAGAGTGTTGGCGATT | 116bp |
| RON5 | ACTCCCACGCTTCTTATCCA | CCATTCTCGTTGACCCACAC | 134bp |
| profilin | GTTGTCCGACCTGAGAAAGGA | CGTAAAGGGCAATGACGATAGAG | 127bp |
| AMA1 | ACAGTCAGGGAATCCTTTGC | TCGCCTTGATGTTGCTATTTT | 116bp |
| M2AP | CCATCAAGAGCCACAACG | GTGAGTCCGAATGCCAAAT | 169bp |
| ROM4 | TCTCAGAGGACGGCTTGTT | AAGGCTGTCGCTGTTTCG | 161bp |
