# Supplementary material for: Variation detection based on next-generation sequencing of type Chinese 1 strains of Toxoplasma gondii with different virulence from China
Source: BMC Genomics. 2015 Oct 30;16:888. doi: 10.1186/s12864-015-2106-z (PMC4628340; doi:10.1186/s12864-015-2106-z)
Supplement: Additional file 2: Table S2. — A: Summary of annotation for SNPs; B: Summary of annotation for indels; C: Summary of annotation for SVs; D: Summary of annotation for CNVs. (DOCX 18 kb) [file 12864_2015_2106_MOESM2_ESM.docx]

Table S2:

A: Summary of annotation for SNPs

| Sample  ID | Upstream | Exonic | | | | Intronic | Splicing | Downstream | Upstream/ downstream | Intergenic | ts | tv | ts/tv | Het  rate (‰) | Total |
| --- | --- | --- | --- | --- | --- | --- | --- | --- | --- | --- | --- | --- | --- | --- | --- |
|  |  | Stop  gain | Stop  loss | Synonymous | Non-  synonymous |  |  |  |  |  |  |  |  |  |  |
| Wh3 | 51930 | 145 | 28 | 55198 | 61706 | 195047 | 42 | 52540 | 4066 | 85096 | 292925 | 212931 | 1.375 | 0.239 | 505856 |
| Wh6 | 51868 | 140 | 28 | 55083 | 61701 | 195498 | 34 | 52552 | 4093 | 84587 | 292740 | 212905 | 1.374 | 0.242 | 505645 |

B: Summary of annotation for indels

| Sample  ID | Upstream | Exonic | | | | | | Intronic | Splicing | Downstream | Upstream/ Downstream | Intergenic | Insertion | Deletion | Het Rate (‰) | Total |
| --- | --- | --- | --- | --- | --- | --- | --- | --- | --- | --- | --- | --- | --- | --- | --- | --- |
|  |  | Stop gain | Stop loss | Frameshift  deletion | Frameshift  insertion | Non-  frameshift  deletion | Non-frameshift  insertion |  |  |  |  |  |  |  |  |  |
| Wh3 | 3269 | 4 | 4 | 136 | 92 | 1358 | 991 | 15884 | 12 | 3791 | 221 | 4094 | 12522 | 17482 | 0.013 | 30004 |
| Wh6 | 3285 | 4 | 4 | 137 | 90 | 1323 | 986 | 16469 | 16 | 3860 | 235 | 4111 | 12793 | 17865 | 0.013 | 30658 |

C: Summary of annotation for SVs

| Sample  ID | Upstream | Exonic | Downstream | Intronic | Upstream/ Downstream | Intergenic | Splicing | INS | DEL | INV | ITX | CTX | Total |
| --- | --- | --- | --- | --- | --- | --- | --- | --- | --- | --- | --- | --- | --- |
| Wh3 | 124 | 479 | 122 | 489 | 33 | 193 | 0 | 668 | 739 | 35 | 95 | 783 | 2320 |
| Wh6 | 415 | 1217 | 404 | 1217 | 50 | 612 | 6 | 3132 | 765 | 25 | 87 | 652 | 4661 |

D: Summary of annotation for CNVs

| Sample  ID | Upstream | Exonic | Intronic | Downstream | Upstream/ Downstream | Intergenic | Duplication  number | Deletion  number | Duplication  length(bp) | Deletion  length(bp) | Total |
| --- | --- | --- | --- | --- | --- | --- | --- | --- | --- | --- | --- |
| Wh3 | 31 | 1730 | 129 | 133 | 15 | 1041 | 85 | 2995 | 282700 | 4940000 | 3080 |
| Wh6 | 15 | 1299 | 31 | 41 | 6 | 550 | 90 | 1852 | 328800 | 7157700 | 1942 |
